# TGF-β and WNT signaling pathways in cardiac fibrosis: non-coding RNAs come into focus

**DOI:** 10.1186/s12964-020-00555-4

**Published:** 2020-06-09

**Authors:** Fatemeh Yousefi, Zahra Shabaninejad, Sina Vakili, Maryam Derakhshan, Ahmad Movahedpour, Hamed Dabiri, Younes Ghasemi, Maryam Mahjoubin-Tehran, Azin Nikoozadeh, Amir Savardashtaki, Hamed Mirzaei, Michael R. Hamblin

**Affiliations:** 1 Department of Genetics, Faculty of Biological Sciences, Tarbiat Modares University, Tehran, Iran; 2 Department of Nanotechnology, Faculty of Biological Sciences, Tarbiat Modares University, Tehran, Iran; 3 Pharmaceutical Sciences Research Center, Shiraz University of Medical Sciences, Shiraz, Iran; 4 Biochemistry Department, Medical School, Shiraz University of Medical Sciences, Shiraz, Iran; 5 Department of Pathology, Isfahan University of Medical Sciences, Isfahan, Iran; 6 Department of Medical Biotechnology, School of Advanced Medical Sciences and Technologies, Shiraz University of Medical Sciences, Shiraz, Iran; 7 Student research committee, Shiraz University of Medical Sciences, Shiraz, Iran; 8 Department of Stem Cell and Development Biology, Cell Science Research Center, Royan Institute for Stem Cell Biology and Technology, ACECR, Tehran, Iran; 9 Department of Pharmaceutical Biotechnology, School of Pharmacy and Pharmaceutical Sciences Research Center, Shiraz University of Medical Sciences, Shiraz, Iran; 10 Student Research Committee, Mashhad University of Medical Sciences, Mashhad, Iran; 11 Department of Medical Biotechnology, Faculty of Medicine, Mashhad University of Medical Sciences, Mashhad, Iran; 12 Pathology Department, School of Medicine,Mashhad Univesity of Medical Sciences, Mashhad, Iran; 13 Research Center for Biochemistry and Nutrition in Metabolic Diseases, Institute for Basic Sciences, Kashan University of Medical Sciences, Kashan, IR Iran; 14 Wellman Center for Photomedicine, Massachusetts General Hospital, Harvard Medical School, 40 Blossom Street, Boston, MA 02114 USA; 15 Laser Research Centre, Faculty of Health Science, University of Johannesburg, Doornfontein, 2028 South Africa

**Keywords:** Cardiac fibrosis, TGF-β/WNT signaling, Non-coding RNAs

## Abstract

Cardiac fibrosis describes the inappropriate proliferation of cardiac fibroblasts (CFs), leading to accumulation of extracellular matrix (ECM) proteins in the cardiac muscle, which is found in many pathophysiological heart conditions. A range of molecular components and cellular pathways, have been implicated in its pathogenesis. In this review, we focus on the TGF-β and WNT signaling pathways, and their mutual interaction, which have emerged as important factors involved in cardiac pathophysiology. The molecular and cellular processes involved in the initiation and progression of cardiac fibrosis are summarized. We focus on TGF-β and WNT signaling in cardiac fibrosis, ECM production, and myofibroblast transformation. Non-coding RNAs (ncRNAs) are one of the main players in the regulation of multiple pathways and cellular processes. MicroRNAs, long non-coding RNAs, and circular long non-coding RNAs can all interact with the TGF-β/WNT signaling axis to affect cardiac fibrosis. A better understanding of these processes may lead to new approaches for diagnosis and treatment of many cardiac conditions.

Video Abstract

Video Abstract

## Introduction

Cardiac fibrosis and abnormal tissue remodeling are pathological findings in many cardiac disorders, such as myocardial infarction, hypertension, myocarditis, cardiac hypertrophy, and dilated cardiomyopathy. These conditions are associated with considerable morbidity and mortality [1–3]. The process of cardiac fibrosis is characterized by a disproportionate accumulation of extracellular matrix (ECM) components. The transformation of cardiac fibroblasts (CFs) to myofibroblasts is the key step in this process, and plays a critical role in the development of fibrosis [4]. When cardiomyocytes die over a period of several days following a sudden cardiac injury, activated myofibroblasts trigger the formation of a fibrotic scar in the affected cardiac muscle [5]. Experimental and clinical evidence has suggested that the transformation of CFs could be regulated by the transforming growth factor beta (TGF-β) and WNT (wingless int1) signaling pathways. Experimental models have shown increased expression of TGF-β and WNT proteins, as key pro-fibrotic factors in cardiac fibrosis [6–8]. Noncoding RNAs (ncRNAs) can be classified into several different types, including small microRNAs (miRNAs or miRs; ~ 22 nucleotides), long noncoding RNAs (lncRNAs; > 200 nucleotides) and circular long noncoding RNAs (circRNAs; > 200 circular nucleotides) [9, 10]. All these ncRNAs have been implicated in the regulation of specific cellular signaling pathways, such as TGF-β and WNT, that act to regulate cytokine secretion and extracellular matrix synthesis [11–13]. A growing body of evidence points to cross-regulation between these two pro-fibrotic pathways mediated via ncRNAs, and its involvement in the pathophysiology of cardiac fibrosis. Understanding these mechanisms will be important to develop new therapeutic agents for treatment of cardiac fibrosis [14–17]. In this review, we will first provide an overview of TGF-β and WNT signaling, and their regulation, followed by a description of their role in the pathogenesis of cardiac fibrosis. Next, we summarize some recent experimental evidence for the involvement of TGF-β and WNT signaling in vascular and cardiac remodeling during fibrosis. Finally, the role of ncRNAs, including miRNAs, lncRNAs, and circRNAs, in TGF-β and WNT signaling in the heart. This is a relatively new field that may provide new avenues for the prevention and treatment of cardiac fibrosis.

## TGF-β signaling

A growing body of evidence indicates that members of the TGF-β family can play a major role in a wide range of processes related to cardiac pathophysiology, including cardiac repair, hypertrophy, fibrotic remodeling, fibroblast activation, and extracellular matrix deposition [18–23]. The TGF-β family is comprised of 33 members, which are multifunctional cytokines interacting with TGF-β receptors to trigger signaling cascade responses in cells [24]. In mammals, the TGF-β isoforms (TGF-β1, β2, and β3) are encoded by three different genes, that are located on separate chromosomes [25–27]. Although in vitro studies have suggested that the three isoforms have similar properties, knockout experiments in mice have shown that each TGF-β isoform exerts distinct effects in tissue development and differentiation in vertebrates [28–31]. TGF-β is initially secreted as a latent complex consisting of three proteins, including TGF-β, latency-associated protein (LAP) and latent TGF-β–binding proteins (LTBPs) so that it is inactive in its latent form. This complex is proteolytically cleaved and activated by an integrin-mediated process [32–34]. Upon binding to their receptor, TGF-βs can activate various processes which can be Smad-dependent (canonical response) or Smad-independent (noncanonical response). The Smad-dependent TGF-β signaling pathway is much better documented. Once activated the TGF-β can bind to heterodimeric receptors (consisting of TGF-β type I and TGF-β type II receptors) and initiate a signaling cascade mediated by the Smad family of transcription factors [35–37]. In the presence of the TGF-β ligand, TGF-β receptor I kinase promotes phosphorylation of the receptor-activated Smads (R-Smads, Smad2 and 3), which mediates the translocation of p-Smad into the nucleus via binding to the common mediator, Smad4 [37]. In the nucleus, the R-Smad/Smad4 complex associates with the transcriptional cofactor p300 and with Fast-1, a nuclear DNA binding protein belonging to the Fast family. This complex binds to DNA, leading to increased expression of the downstream effectors of the TGF-β signaling pathway [38, 39]. There are fundamental differences between the processes mediated by Smad2 and Smad3. Smad2 is required for normal development processes, whereas Smad3 is required for TGF-β-induced gene expression. Microarray analysis in adult Smad3^−^/^−^ and Smad3^+^/^+^ mice revealed that TGF-β did not induce any transcription in Smad3^−^/^−^ fibroblasts [40]. Intriguingly, in a recent report, Smad2 homozygous mice (Smad2^−^/^−^) were not viable during embryogenesis, suggesting that Smad2 is required for normal development [41]. Two inhibitory Smads (I-Smad), named Smad6 and Smad7, do not allow R-Smad phosphorylation, and prevent subsequent nuclear translocation of R-Smad/Smad4 heterocomplexes, because they compete for the normal binding of Smad2 and Smad3 to the TGF-β R1 [42]. After completion of the Smad transcriptional activity, coactivator p300 facilitates an interaction between Smad3 and the E3 ligase complex causing Smad3 ubiquitination, leading to its degradation by the ubiquitin-proteasome pathway [43, 44].

In addition, TGF-β can also result in direct activation of a non-canonical response via three different pathways, namely the PI3K/Akt, RhoA-ROCK axis and MAPK cascades. In the PI3K/Akt pathway, activated TGF-β type I receptor stimulates a signaling cascade involving phosphatidylinositol-3-kinase (PI3K) and protein kinase B (also known as Akt). In this pathway, PI3K activation mediates phosphorylation and activation of Akt kinase, which in turn modulates several downstream effectors, such as the mammalian target of rapamycin (mTOR), glycogen synthase kinase-3β (GSK-3β) and many others [45, 46]. The second pathway involves Rho-associated protein kinases (ROCKs) which are coiled-coil-containing protein kinases existing in two isoforms, ROCK1, and ROCK2 [47]. Experiments have confirmed the importance of ROCKs in regulation of various cellular functions, especially cardiac fibrogenesis [48, 49]. It has been demonstrated that inhibition of ROCK prevented cardiac fibrosis in response to transverse aorta constriction (TAC) and myocardial infarction (MI) [50]. In the third pathway, mitogen- activated protein kinases (MAPKs) transmit the signal from the cell membrane to the nucleus, and regulate gene expression through extracellular signal-regulated kinase 1 and 2 (Erk1/2 or p44/42), c-Jun N-terminal kinases (JNKs), and the p38 isoforms (α, β, γ, and δ) [51]. The Smad-independent activation of MAPKs was confirmed by a study that found that activation of MAPKs occurred in TGF-β treated cells, even in the presence of Smad4-deficient or dominant-negative Smads [52].

## WNT signaling

Several publications have indicated the importance of the WNT signaling cascade in normal development and the pathogenesis of many diseases [53–56]. WNT ligands are highly conserved secreted glycoproteins, which are transcribed from 19 genes in mammals, and are subdivided into 12 conserved subfamilies [57]. A family of seven transmembrane receptors called Frizzled (Fz) act as receptors for WNT ligands [58]. WNT ligands engage the Fz receptors and the low-density-lipoprotein-receptor-related proteins (LRP)5 and LRP6 act as co-receptors resulting in the formation of a complex at the plasma membrane. Activated Fz receptors activate not only the β-catenin-dependent pathway, but also trigger a number of β-catenin-independent signaling cascades.

In the WNT/β-catenin signaling pathway, the activated Fz/LRP complex activates the Dishevelled (DVL) protein, which interacts with a “destruction complex” comprised of axin, adenomatous polyposis coli (APC), glycogen synthase kinase 3 (GSK3), casein kinase 1 (CK1), plus the ubiquitin ligase, β-TrCP. This destruction complex normally causes the ubiquitination and consequent destruction of β-catenin [59–61]. When the destruction complex binds to DVL it is inhibited, and β-catenin escapes from ubiquitination and degradation [62, 63]. Eventually, the stabilized β-catenin translocates to the nucleus where it activates WNT-responsive genes, by binding to T-cell factor/lymphoid enhancer-binding factor-1 (TCF/Lef-1) transcription factors, and other co-factors, such as p300 and CREB binding protein (CBP) [56].

In the β-catenin-independent signaling pathway, WNT4, WNT5a or WNT11 ligands can stimulate the Fz receptor to trigger gene transcription, by activating the planar cell polarity pathway, and a calcium-dependent pathway. Signal transduction through the WNT/calcium cell polarity pathway typically consists of protein kinase C (PKC), calmodulin kinase II and calcineurin. Calcineurin is a Ca^2 + −^sensitive enzyme activated by Ca^2+^ release which results in elevated nuclear levels of the AP-1/c-Jun transcription factor [64, 65]. In the planar cell polarity pathway, certain MAPKs (JNK and ERK1/2 kinases) and the RhoA-ROCK axis act as important regulators to trigger signaling. In this pathway, WNT proteins activate Rho signaling and Jun N-terminal kinase (JNK) through DVL, leading to the modulation of cellular activity and polarity via ATF/CREB activation [66].

Endogenous WNT antagonists, such as the Dikkopf (DKK) and secreted frizzled-related protein (sFRP) families can regulate WNT signaling [67–69]. Many studies have emphasized the key role of the WNT signaling pathway in cardiac fibrosis, and it has been suggested that regulation of signaling pathways might be a useful pharmacological target for treatment of cardiac disease [70–72].

## Crosstalk between the TGF-β and WNT signaling pathways

Recent studies have highlighted the extensive cross-talk between the TGF-β and WNT pathways, which could be responsible for the transcription of pro-fibrotic genes. These pathways could create a positive feedback loop or a negative feedback loop that impacts on the transcriptional activity of other signaling cascades. For instance, loss of the WNT co-receptor LRP5, in bleomycin-induced lung fibrosis decreased the expression of TGF-β1, and attenuated the induction of fibrosis [73]. Consistently, over-expression of a constitutively active TGF-β receptor type I in a mouse model (Ad-TBRI^act^-induced fibrosis) enhanced the stability and nuclear accumulation of β-catenin in primary cultured fibroblasts. These results showed that TGF-β activated the canonical WNT pathway through a decrease in Dkk-1 via MAPKp38, and this could be a major molecular mechanism for the activation of the canonical WNT pathway [6]. In addition, it was reported that sirtuin 3 (SIRT3) can increase the enzymatic activity of GSK3, resulting in the blockage of TGF-β expression in tissue fibrosis. The SIRT3 deficiency induced GSK3β hyperacetylation resulting in reduced GSK3β activity and lower phosphorylation of Smad3, and β-catenin activation. These events lead to stabilization and activation of Smad3 and β-catenin transcription factors involved in the tissue fibrosis process [74]. In addition, upon TGF-β stimulation, the Axin, WNT scaffold protein, can promote Smad7 degradation by forming a complex with Smad7 and the E3 ubiquitin ligase Arkadia [75]. Another report revealed that Axin facilitated Smad3 binding to the type I receptor to promote the tail-phosphorylation of Smad3, which induced the transcription of pro-fibrotic genes [76].

Furthermore, the microRNA, miR-29 inhibited the synthesis of ECM caused by TGFβ1, through inhibition of WNT3a/β-catenin signaling pathway in human orbital fibroblasts [77]. Yeast two-hybrid and GST-pull down assays showed a physical interaction between DVL-1 and Smad3. Stimulation of the TGFβ pathway leads to an increase in DVL-1, and Smad3 binding in vivo [78]. Co-treatment with both WNT-3a conditioned medium, and TGFβ led to enhancement of nuclear β-catenin, whereas TGFβ alone had no effect. Moreover, Smad3 over-expression enhanced the ability of WNT-3a to increase transcription, suggesting that Smad3 is required for the effects of TGF-β on gene transcription [79]. The ability of TGF-β to activate the canonical WNT signaling pathway, and the functional impact of this mechanism on fibrotic processes has been reported in many organs, as well as the heart [6, 80–82]. In summary, these data point to the important role of TGF-β signaling in the activation of the β-catenin-dependent pathway, and conversely, the role of the WNT/β-catenin signaling pathway to trigger TGF-β signaling. Taken together, it appears that mutual co-activation of these two pathways is required to trigger the actual fibrotic response.

## Cardiac fibrosis

Cardiac fibrosis is characterized by the increased activity of cardiac fibroblasts, resulting in the accumulation of ECM proteins (e.g. collagen I and III), which produce increased myocardial stiffness, and thereby increase the risk of heart failure and sudden cardiac death [83]. Cardiac fibrosis commonly occurs in several types of cardiovascular disease, such as those caused by diabetes, ischemia, aging, and inherited cardiomyopathy, which cause considerable morbidity and mortality [1–3]. Healthy heart tissue consists of endothelial cells, vascular smooth muscle cells, fibroblasts, and myocytes [84–86]. The alteration of the collagen matrix results from dysregulation of the balance between pro-fibrotic and anti-fibrotic factors, including cytokines, chemokines, hormones, growth factors, and proteases. This dysregulation causes a swing in the balance, leading to excess synthesis or inhibition of degradation [87]. Fibroblasts are involved in collagen formation and degradation, and any derangement in this process leads to collagen expansion and myocardial fibrosis [88]. The key event in cardiac fibrosis is the transformation of cardiac fibroblasts (CFs) into myofibroblasts, which are involved in ECM production, and accelerating the fibrotic process following cardiac injury. After cardiac ischemia, cardiomyocytes gradually die over a period of several days starting after myocardial infarction, and then myofibroblasts replace the dead cells, and largely produce the fibrotic scar [5]. There are two main stages in myofibroblast transformation: in the first stage, small adhesion complexes and stress-fiber network formation occurs to develop proto-myofibroblasts from fibroblasts, and to facilitate the migration of these cells into the injured tissue. In the second stage, these proto-myofibroblasts convert to mature myofibroblasts which secrete alpha-smooth muscle actin (α-SMA) and cadherin-11 by approximately 20–30 h following cardiac injury [89–91]. Activated myofibroblasts deposit large amounts of ECM proteins in the infarcted area [92]. In cardiovascular disease, myocardial fibrosis can be divided into three stages: mild diffuse fibrosis, severe diffuse fibrosis, and segmental fibrosis.

Finding an effective treatment for myocardial fibrosis is a major clinical challenge, which may dramatically improve the survival rate and the quality of life in patients. Cardiac magnetic resonance (CMR) imaging has been used to evaluate the extent of diffuse myocardial fibrosis, and for monitoring patients with cardiac fibrosis [93–95]. In one study by McCrohon and colleagues, myocardial fibrosis was assessed in patients suffering from dilated cardiomyopathy (DCM) using gadolinium-CMR demonstrating high sensitivity, specificity, and good spatial resolution. The results suggested that approximately one third of patients with DCM may have myocardial scarring or fibrosis [96]. Therefore, CMR imaging techniques may be used to delineate foci of fibrosis in patients with DCM. Myocardial fibrosis is associated with electrical dysfunction that leads to ventricular arrhythmias in tricuspid atresia patients [97]. Ventricular myofiber disorganization and interstitial fibrosis have been demonstrated in patients with tetralogy of Fallot [98]. A study revealed increased macroscopic fibrosis in the left ventricles (LV) of deceased patients with congenital aortic stenosis and coarctation [99]. Also, LV fibrosis has been detected in patients with idiopathic DCM who only had mild symptoms. The data suggested that collagen accumulation might be responsible for the impaired LV diastolic function found in these patients [100]. Various alterations in molecular pathways and cellular effectors have been shown to occur in various cardiac fibrotic conditions, and it is important to assign the relative contribution(s) of each pathway, and the therapeutic implications. Therefore, the effectiveness of anti-fibrotic strategies requires a better understanding of the mechanisms of cardiac fibrosis, which may depend on the underlying etiology, severity and extent of disease.

### The role of TGFβ and WNT signaling in cardiac fibrosis

**Fig. 1:**
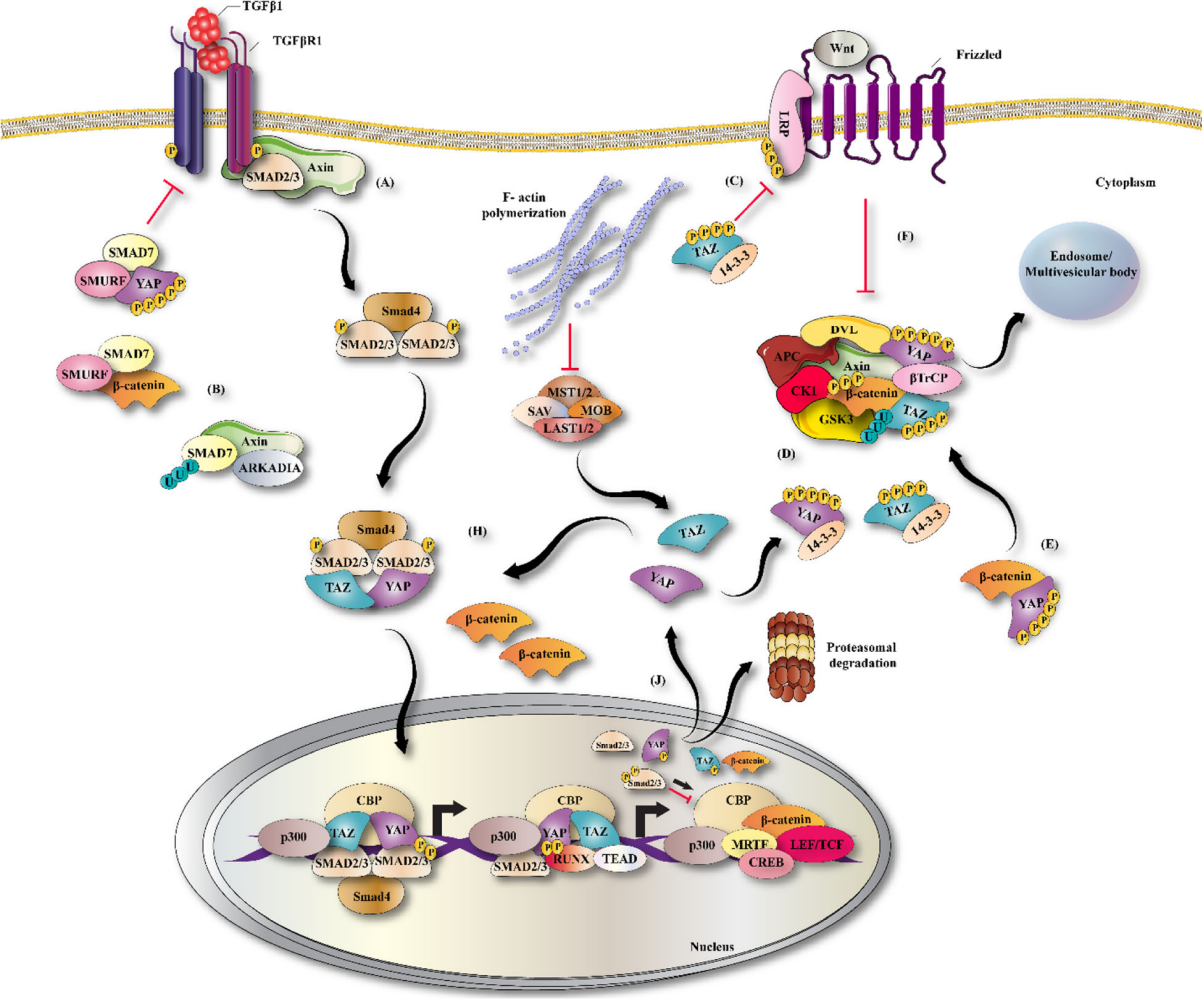
Role of WNT/TGFβ signaling pathways in pathogenesis of cardiac fibrosis. Simplified scheme showing the WNT/β-catenin pathway, and Smad-dependent TGF-β signaling are involved in the stimulation of myofibroblast proliferation and differentiation, thus promoting fibrogenesis

Myofibroblasts expressing expressed α-SMA (smooth muscle actin) are mainly involved in the excessive synthesis and degradation of collagen in cardiac fibrosis [4, 101]. Following a cardiac injury, fibroblasts transdifferentiate into myofibroblasts, which are normally absent in the healthy heart. Myofibroblasts are in between a fibroblast and a smooth muscle cell in phenotype [102, 103]. Myofibroblasts mainly secrete ECM proteins, such as periostin, collagens I and III, and fibronectin, and also a number of cytokines which regulate the inflammatory response at the injury site [89]. In heart disease, fibrotic remodeling associated with cellular and molecular mechanisms, leads to disturbance of cardiac function in different ways. Myocardial fibroblasts are stimulated both mechanically and chemically to undergo differentiation into the myofibroblast phenotype [104]. The TGF-β and WNT signaling pathways are two key regulators of myofibroblast biology in cardiac fibrosis [56, 105–107]. High expression levels of TGF-β1 (a prototypical fibrogenic cytokine) has been reported during cardiac fibrosis both in humans and experimental models [108, 109]. The increase in TGF-β causes nuclear accumulation of Smad2/3 in myofibroblasts, and decreases the inhibitory Smad6 and Smad7, thereby inducing the activation of numerous pro-fibrotic genes [21, 110, 111]. The resistance of fibroblasts isolated from Smad3 −/− mouse embryos to TGF-β1 induction of ECM proteins, confirmed that Smad3 mediated TGF-β transactivation of these ECM promoters [40, 112]. In the injured heart, the Smad3 signaling cascade may be the first step in myofibroblast differentiation by promoting α-SMA transcription [113]. In α-SMA-positive myofibroblasts, TGFβ1 could up-regulate matrix proteins (such as ED-A fibronectin) and increase the deposition of collagen sub-types (such as collagens I, III, and VI) by regulating the levels of plasminogen activator inhibitor (PAI)-1 and tissue inhibitor of metalloproteinases (TIMPs), and also by regulating the levels of other pro-fibrotic cytokines [114–116]. Collagen type VI plays a role in cardiac remodeling by increasing myofibroblast differentiation, whereas collagen types I and III stimulate the proliferation of CF via increasing ERK1/2 activity [117]. WNT/β-catenin signaling is enhanced in areas of scar formation, and in epicardial activation in mouse models [118]. Activation of the WNT/β-catenin pathway by TGF-β is well-documented. TGF-β increases Akt phosphorylation through PI3K activation, thus inactivating GSK3β (an enzyme involved in β-catenin degradation), which promotes cardiac fibrosis [119]. In recent years, a number of reports have indicated that the canonical WNT/β-catenin pathway, and Smad-dependent TGF-β signaling are involved in the stimulation of myofibroblast proliferation and differentiation, thus promoting fibrogenesis [120, 121]. WNT3a can promote migration in cultured fibroblasts, as well as the adoption of a myofibroblast-like phenotype, in part by up-regulation of TGF-β signaling through Smad2. WNT3a-induced α-SMA expression was reversed following knockdown of β-catenin, suggesting that fibrosis was dependent on canonical WNT signaling through β-catenin [14]. GSK-3β exerts a novel and central role in regulating myocardial fibrotic remodeling via modulation of canonical TGF-β1 signaling through direct interaction with Smad-3. Moreover, genetic and pharmacological approaches have demonstrated that inhibition of GSK-3β can induce the transformation of fibroblasts to myofibroblasts [122]. In cardiac fibroblasts, over-expression of FGF23 promoted fibroblast proliferation through activation of β-catenin signaling, leading to TGF-β up-regulation [15]. In primary cardiac fibroblasts, lack of endogenous sFRP-1 increased αSMA and collagen expression, and promoted differentiation of fibroblasts into myofibroblasts [71]. Over-expression of sFRP2 significantly reduced ventricular fibrosis, and improved cardiac function in vivo [123]. On the other hand, sFRP2 antibody therapy decreased apoptosis and fibrosis, and improved cardiac function in a hamster model of heart failure [124]. In agreement with this, mice lacking the sFRP2 gene that were subjected to myocardial infarction, exhibited a reduction in cardiac fibrosis [125]. These findings suggest that sFRPs have multiple modes of action in fibrotic processes. In a WNT1 Cre transgenic mouse model subjected to myocardial ischemia, WNT 1 expression occurred within 2 days following ischemia, with high expression levels found in both epicardial cells and cardiac fibroblasts. Experimental models using CF-specific loss of β-catenin, or exogenous over-expression of WNT1, have suggested that canonical WNT signaling can induce collagen gene expression during cardiac fibrosis. Chromatin immunoprecipitation (ChIP) assays with an anti-β-catenin antibody, showed that the β-catenin/LEF/TCF transcriptional complex directly bound to ECM genes in CFs [126]. In agreement with these studies, Dickkopf-3 (DKK3), a WNT signaling pathway inhibitor, attenuated cardiac fibrosis in cardiac hypertrophy induced by administration of angiotensin II (AngII), by promoting AngII degradation. DKK3 inhibited the phosphorylation of the metalloproteinase enzyme known as “a disintegrin and metalloproteinase 17” (ADAM17), which in turn increased angiotensin converting enzyme 2 expression, and subsequently increased AngII degradation. AngII degradation was triggered by the inhibition of GSK-3β and β-catenin, and the decreased translocation of β-catenin to the nucleus. On the other hand, DKK3 knockdown by siRNA achieved the opposite effects [127]. The blocking of the TGFβ and WNT pathways prevented the development of fibrosis in animal models, but whether these two signaling pathways contribute to chronic pathologic fibrosis in humans is still unknown. Figure 1**and** Table 1 illustrate the WNT and TGFβ signaling pathways which have been associated with the pathogenesis of cardiac fibrosis.

**Table 1:** TGFβ and WNT signaling pathways involved in the regulation of cardiac fibrosis

Pathway	Function	model	Anti-fibrotic or pro-fibrotic)	Reference
Aldehyde dehydrogenase-2 (ALDH2)	Decreased β-catenin, phosphorylated GSK-3β, and WNT-1	MI/rat	Anti	[128]
WNT10b	Increased Axin2, Lef1 and Tcf7	Transgenic (TG) WNT10b mice	Anti	[129]
S100A4	Decreased β-catenin and phosphorylated β-catenin	LAD /mouse; CFs	Pro	[130]
WNT3a and WNT5a	Decreased glycogen synthase kinase 3β (GSK3β)	Human CFs	Pro	[131]
Qishen Granule (QSG)	Inhibition of the TGF-β/Smad3 pathway and the phosphorylation of GSK-3β	HF/Rat	Anti	[132]
Xinfuli Granule (XG)	Decreased Smad3, P-Smad3 and Smad2 protein	MI/rat	Anti	[133]
Human antigen R (HuR)	Increased TGF-β1	TAC/HuR-deletion mouse	Pro	[134]
Transient receptor potential ankyrin 1 (TRPA1)	Decreased TGF-β, IL-4 and IL-10	TAC/mouse	Pro	[135]
Small molecule inhibitor ICG-001	Decreases β-Catenin	Ang II infusion/ Cfs Rat	Anti	[136]
EphrinB2 (erythropoietin-producing hepatoma interactor B2)	Increased TGF-β/Smad3 pathway and STAT3	Ang II MI/ Mouse	Pro	[137]

## Non-coding RNAs

ncRNAs play important roles in many cellular processes. Based on nucleotide length, they are classified into: small miRNAs (22 nucleotides) and lncRNAs (> 200 nucleotides) [138, 139]. Circular RNAs (circRNAs) are a new type of ncRNAs that can regulate gene expression and protein production by acting as an miRNA sponge [140–142]. These three groups of ncRNAs can be detected in blood and other body fluids, such as urine and breast milk, and can act as powerful tools for detection and therapy of cardiac diseases, including cardiac fibrosis [4, 50, 143]. The function of ncRNAs during cardiac remodeling, involves them being secreted from some cells, and then being actively taken up by other target cells, thus contributing to cell–cell communications and paracrine signaling pathways [144–148].

### The role of ncRNAs in cardiac fibrosis

The role of ncRNAs (especially miRNAs) has been shown in many studies [149–152]. The down-regulation of miR-122 has been reported in endomyocardial biopsies from patients with severe aortic valve stenosis (AS), compared to control subjects. Experiments in human fibroblasts have shown that miR-122 might be involved in myocardial fibrosis [153]. In one recent study, miR-34a was suggested to be a positive regulator of fibrogenesis. miR-34a was dynamically up-regulated following myocardial infarction, and inhibition of miR-34a resulted in decreased severity of cardiac fibrosis in mice. Functionally, miR-34a over-expression increased the pro-fibrogenic activity. miR-22 is also involved in regulation of cardiac fibrosis. miR-22 was significantly reduced following myocardial infarction, leading to increased collagen deposition, thereby promoting cardiac fibrosis. Some ECM proteins, including Col1a1 and Col3a1, were over-expressed after miR-22 knock-down in cultured CFs. Another study demonstrated that miR-29 levels were decreased under cardiac stress, thereby increasing collagen expression and promoting cardiac fibrosis [154]. Over-expression of several lncRNAs, including n379599, n379519, n384648, n380433, and n410105, increased the expression of Col8A1, Col3A1, and FBN1 and increased cardiac fibrosis by inducing phosphorylation of Smad2/3 and TGF-β signaling. Silencing of these lncRNAs induced the opposite effect [12]. The over-expression of miR-1954 showed a reduction in cardiac mass and blood pressure in mice. It also reduced expression of cardiac fibrotic genes, inflammatory genes, and hypertrophy marker genes. They found that miR-1954 played an important role in cardiac fibrosis by targeting THBS1, therefore it could be a promising strategy for the treatment of cardiac fibrosis [155].

CircRNAs are another type of single-stranded RNA molecules, which are involved in many normal physiological processes, as well as the pathogenesis of cardiovascular disease [156, 157]. For example, circHIPK3 affected the proliferation and migration of CFs, and the expression of genes such as COL1A1, COL3A1, α-SMA through “sponging up” miR-29b-3p. Silencing of circHIPK3 showed opposite effects on CF proliferation, and the diastolic function was improved in vivo [158]. Microarray analysis of several circRNAs revealed a significant up-regulation of circRNA_010567 in a diabetic db/db mouse model compared to controls. Knockdown of circRNA_010567 reduced the synthesis of fibrotic proteins, such as Col I, Col III and α-SMA in CFs, which had been treated with Ang II [13]. ncRNAs could be attractive candidates as putative biomarkers for cardiac fibrosis in a variety of cardiovascular conditions, although their function needs to be fully investigated in future studies.

### Role of ncRNAs in TGFβ and/or WNT signaling

The relationship between ncRNAs and TGFβ and/or WNT signaling has been demonstrated in studies, and could affect many pathogenic conditions. Colorectal cancer patients with high expression of certain lncRNAs had a shorter overall survival and a worse response to chemotherapy. It was found that these lncRNAs promoted CRC progression by activating Wnt/ β -catenin pathway through activator protein 2 α. Furthermore, lncRNA can induce multidrug resistance through activating Wnt/ β-catenin signaling by up-regulating MDR1/P-gp expression [159].

Wu et al. showed that down-regulation of lncRNA CCAT2 reduced the expression of TGF-β, Smad2 and α-SMA in breast cancer patients. CCAT2 promoted growth and metastasis of breast cancer by regulating the TGF-β signaling pathway [160].

Shan et al. found that over-expression of lncRNA Linc00675 inhibited the proliferation and migration of colorectal cancer cells. Furthermore, they found that the expression of miR-942 in clinical colorectal cancer tissues was higher than in normal tissue. More importantly, the inhibitory effect of Linc00675 was also attenuated by a miR-942 mimetic, suggesting that down-regulation of miR-942 represented one of the mechanisms by which Linc00675 inhibited the proliferation and metastasis of colorectal cancer. They demonstrated the inhibition of Wnt/β-catenin signaling in the Linc00675/miR-942 regulated pathway in colorectal cancer cells [161].

Yoan et al. found that over-expression of lncRNA CTD903 inhibited invasion and migration of colorectal cancer cells by repressing Wnt/β-catenin signaling and predicts favorable prognosis [162]. *LncTCF7* is a lncRNA which required for liver cancer stem cell self-renewal and tumor progression. *lncTCF7* recruited the SWI/SNF complex to the promoter of *TCF7* to regulate its expression, leading to activation of Wnt signaling. *lncTCF7*-mediated Wnt signaling primes liver cancer stem cell self-renewal and tumor propagation [163].

In addition to lncRNAs, microRNAs have important roles in TGFβ and/or WNT signaling. Yu et al. found that microRNA-21 induces stemness by down-regulating TGF-β receptor 2 (TGFβR2) in colon cancer cells [164]. Tan et al. studied human orbital fibroblasts to show that TGFβ1 treatment decreased miR-29 expression, which could inhibit TGFβ1. MiR-29 inhibited TGFβ1-induced proliferation and decreased colony formation of orbital fibroblast cells after TGFβ1 treatment. MiR-29 mediates TGFβ1-induced extracellular matrix synthesis through activation of Wnt/β-catenin pathway in human orbital fibroblasts [77].

In another study, salvianolic acid B (Sal B) treatment induced the inactivation of the Wnt/β-catenin pathway, with an increase in phosphorylated-β-catenin and Wnt inhibitory factor 1. It was found that miR-17-5p was reduced in vivo and in vitro after Sal B treatment. As confirmed by luciferase activity assays, WIF1 was a direct target of miR-17-5p. Importantly, the suppression of HSCs induced by Sal B was almost completely inhibited by miR-17-5p mimetics. Therefore, miR-17-5p activates Wnt/β-catenin pathway to result in HSC activation through inhibiting WIF1 expression [165].

## The relationship between ncRNAs and TGFβ and/or WNT signaling in cardiac fibrosis

**Fig. 2:**
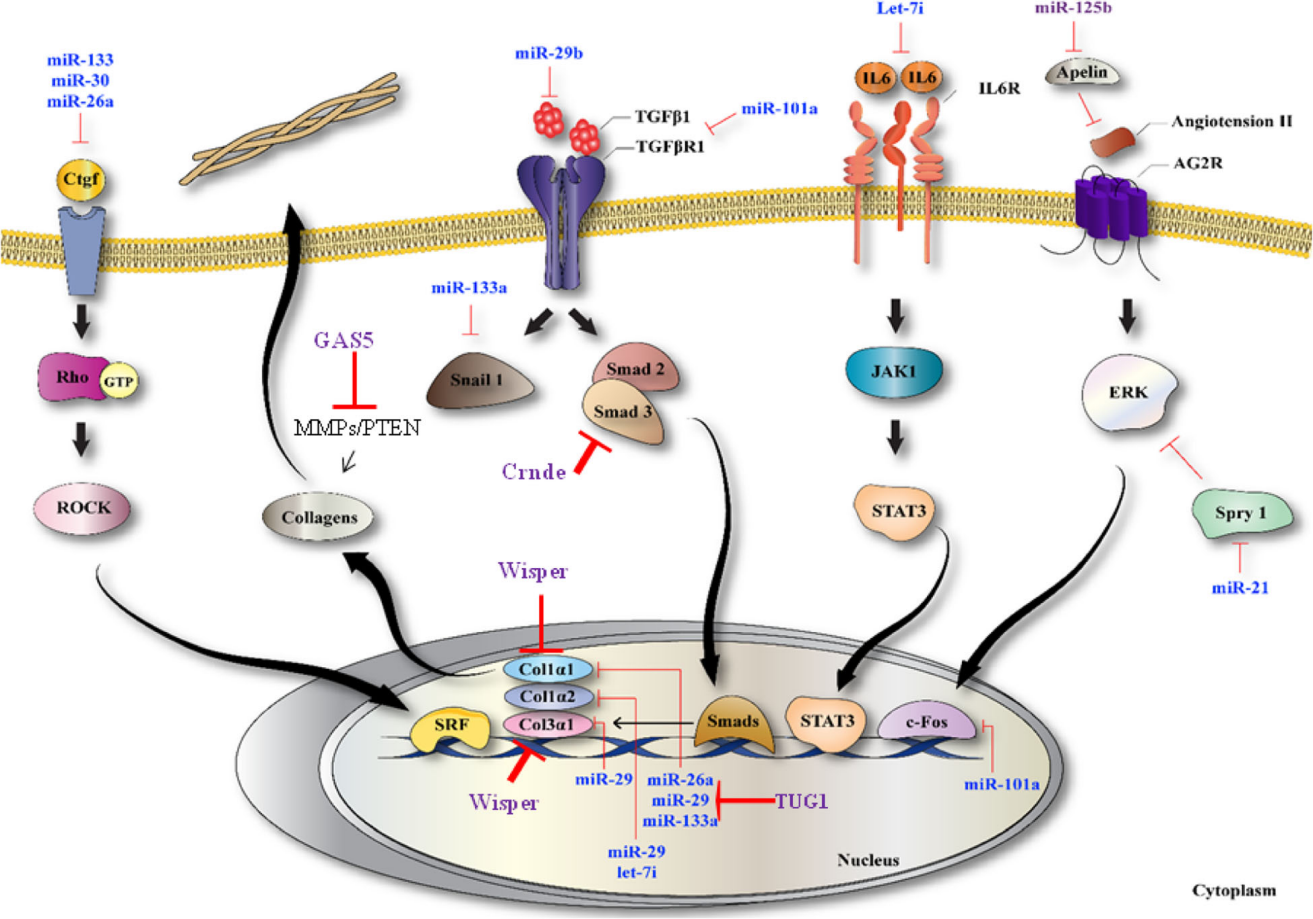
The crosstalk between microRNAs and WNT/TGFβ signaling pathways in cardiac fibrosis. Schematic representation that shows microRNAs affect cardiac fibrosis progression by targeting WNT/TGFβ signaling pathway associated proteins

Many studies have provided evidence for cross-talk between fibrosis development and miRNA deregulation, via the TGFβ and WNT signaling pathways (Fig. 2). Some of these studies are summarized in this section (Tables 2, 3 and 4).

**Table 2:** miRNAs involved in the regulation of cardiac fibrosis mediated by TGFβ/WNT signaling pathways

miRNAs	Expression (up/down)	Function	model	Anti- fibrotic Pro-fibrotic	Reference
miR-378	Down	Activate RTK, GRB-2/TGFβ	AngII, TAC/mouse; CFs	Anti	[179]
miR-101a	Down	Suppress TGFβ receptor I, p-Smad3	MI, hypoxia/rat	Anti	[180]
miR-145	Up	Suppress TGFβ receptor II	Smooth muscle cells; Ang II/mouse	Anti	[181]
miR-675	Down	Suppress TGFβ receptor I	TGFb /mouse CFs	Anti	[182]
miR-10a	Up	Activate TGF-β1/Smads signaling pathway	Rat CFs	Pro	[183]
miR-15	Up	Suppress TGFβ receptor I, p38, endoglin, Smad3/7	TAC/mouse	Anti	[184]
miR-9	Down	Suppress TGFβ receptor II	High glucose/human CFs	Anti	[185]
miR-223	Up	Suppress RASA1 /Activate RAS and smad signaling pathways	MI/ Rat CFs	Pro	[186]
MiR-323a-3p	Up	Suppress TIMP3/ActivateTGF-β pathway	AngII, TAC/mouse; CFs	Pro	[187]
miR-202-3p	Down	Suppress TRPM6, TGFβ1, Smad2 and p- Smad2	Rat myocardial ischemic-reperfusion (I/R) injury	Anti	[188]
miR-433	Up	Activate TGFβ1, ERK, p38 kinase and Smad3	MI/mice	Pro	[189]
miR-29b	Down	Suppress Smad3 signaling	MI/Rat	Anti	[190]
miR-495	Down	Suppress NOD1, NF-κB and TGFβ1/Smad signaling pathways	High glucose/human CFs	Anti	[191]
miR-154	Up	Suppress GSK-3β/ Activate WNT signaling	Human CFs	Pro	[192]
miR-154	Up	Suppress DKK2/ Activate WNT signaling	Human CFs	Pro	[193]
miR-199a	Up	Suppress secreted frizzled-related protein 5 (SFRP5)	ISO, Rat CFs	Pro	[194]
miR-503	Up	Activate connective tissue growth factor (CTGF) and TGF-β	AngII, TAC/mouse; CFs	Pro	[195]

**Table 3:** LncRNAs involved in the regulation of cardiac fibrosis mediated by TGFβ/WNT signaling pathways

LncRNAs	Expression (up/down)	Function	Model	Anti- fibrotic or Pro-fibrotic	Reference
n379519	Up	Sponged miR-30/Activated TGFβ signaling pathway	TGFβ MI/ Rat CFs	Pro	[196]
Taurine Upregulated Gene 1 (TUG1)	Up	Sponged miR-29c/Activated TGFβ signaling pathway	Congenital human heart tissue, chronic hypoxic mouse CFs	Pro	[197]
Homeobox A11 antisense (HOXA11-AS)	Up	Activated TGFβ signaling pathway	Mouse CFs	Pro	[198]
Colorectal neoplasia differentially expressed (Crnde)	Down	Inhibited the binding of Smad3 to the α-SMA gene promoter via interacting with rSBEs	DCM/Human, mouse/CFs	Anti	[199]

**Table 4:** Circular RNAs involved in the regulation of cardiac fibrosis mediated by TGFβ/WNT signaling pathways

CircRNAs	Expression (up/down)	Function	model	Anti- fibrotic Pro-fibrotic	Reference
circRNA_010567	Up	Regulated TGF-β signaling and ECM synthesis via sponging up miR-141	Diabetic mice myocardium and CFs	Pro	[178]
CircACTA2	Up	Regulated the expression and function of α-SMA, by acting as a decoy for miR-548f-5p. TGF-β over-expression increased circACTA2.	Rat, mouse, and human VSMCs	Pro	[13]

For example, researchers found that TGF-β1 and miR-21 expression were up-regulated in the border zone of mouse heart after myocardial infarction, whereas TGF-β receptor type III was down-regulated. TGFβRIII is a negative regulator of the TGF-β pathway. Over-expression of miR-21 attenuated TGFβRIII expression in cardiac fibroblasts, thereby potentially contributing to excessive ECM production via the TGF-β pathway [166]. However, using miR-21–knockout mice and knock-down of miR-21 expression, showed that cardiac fibrosis could still develop under conditions of cardiac stress. These data suggested that miR-21 was not essential for pathological cardiac remodeling [167]. In animal models of myocardial infarction, miR-101 is usually repressed, and delivery of miR-101 by an adeno-associated virus led to amelioration of the infarcted heart through activation of the proto-oncogene c-fos and silencing of the TGFβ1 pathway [168]. miR-133a was down-regulated in a model of atrial fibrillation in dogs, induced by administration of nicotine. Over-expression of miR-133a decreased TGF-β1 and levels of TGF-β receptor type II, and also reduced the collagen content in cultured atrial fibroblasts [169]. AS mentioned above, miR-34a over-expression increased the pro-fibrogenic activity, whereas miR-34a inhibition reduced the activity of TGF-β1 by directly targeting Smad4 in CF cells [11]. Furthermore, it is suggested that direct down-regulation of TGFβ by miR-22 may have mediated the anti-fibrotic effect. miR-29 may also play a role in the regulation of cardiac fibrosis. In a mouse model of aortic aneurysm, inhibition of miR-29 led to greater stability in the aortic walls, and prevented rupture by increasing the expression of several ECM genes, such as Col1A1, Col3A1, and elastin [170]. It is indicated that TGFβ could repress miR-29 expression under cardiac stress [154]. Suppression of miR-155 reduced infarct size, improved LV function, and attenuated collagen deposition in vivo. Knockout of miR-155 arrested the proliferation of CFs and their differentiation into myofibroblasts, through up-regulation of “tumor protein p53-inducible nuclear protein1” (TP53INP1) [171]. TP53INP1 is a pro-apoptotic protein that can interact with p53 and modulate p53 transcriptional activity [172]. A previous report showed that TGF-β could indirectly regulate TP53INP1 expression by affecting miR-155 levels in liver cancer cells [173]. miR-24 also plays a role in the regulation of myocardial infarction-induced cardiac fibrosis, possibly through targeting furin [174]. Furin is a protease related to the TGF-β pathway, and has been reported to regulate production of collagen in fibrosis [175]. A different study revealed that up-regulation of a LncRNA, also known as “myocardial infarction associated transcript” (MIAT), in myocardial infarction was accompanied by down-regulation of miR-24 and up-regulation of Furin and TGF-β1 [176]. One lncRNA, known as HOTAIR (HOX transcript antisense RNA) was associated with myocardial fibrosis via activation of the WNT signaling pathway through targeting the URI1 gene (unconventional prefoldin RPB5 interactor 1). HOTAIR over-expression elevated the expression of axin2, β-catenin and p-GSK-3β, and also promoted cell proliferation and migration in CFs. Results showed that the regulatory effects of HOTAIR over-expression on CF functions were the same as those found after URI1 over-expression, suggesting that HOTAIR may regulate CFs by targeting URI1 [12].

Seo et al. showed that miR-384-5p was a key mediator in the formation of a transactivation circuit between the TGF-β and WNT signaling pathways in cardiac fibrosis. This function might be related to the modulation of different receptors, such as Fzd1, Fzd2, TGFβR1, and LRP6. The expression of miR-384-5p was significantly decreased in CFs at 3 and 7 days after ischemia/reperfusion (I/R) injury, and 24 h after TGF-β treatment. Moreover, the over-expression of miR-384-5p significantly attenuated cardiac fibrosis and decreased the fibrotic area in the rat I/R injury model. These findings suggested that miR-384-5p might be able to control cardiac fibrosis [177]. A recent study reported that inhibition of miR-27a exhibited a cardio-protective effect by regulating the WNT and TGF-β pathways, through modulating the expression of the β-catenin, p-GSK-3β and α-SMA genes. According to this investigation, miR-27a levels were increased in the fibrotic heart tissue of rats with chronic heart failure (CHF), and the data suggested that miR-27a might be a target for treating cardiac fibrosis [17]. Furthermore, circRNA_010567 regulated TGF-β signaling and ECM synthesis via sponging up miR-141 [178]. Moreover, it has been reported that CircActa2 was involved in myocardial and endocardial fibrosis, through regulation of the expression and function of α-SMA, by acting as a decoy for miR-548f-5p. It was shown that TGF-β over-expression increased circACTA2, thereby facilitating the formation of stress fibers and cell contraction [13].

## Conclusions

The relentless progression of fibrosis is well known to be a pathological finding in many cardiac conditions. The mechanisms responsible for this process are rather complex, and involve multiple pathways. Therefore, a better understanding of the functional characteristics and molecular profiles of these fibrotic processes could provide solutions for the prevention and treatment of fibrotic lesions in the heart. Accumulating evidence points to the cross-talk between the TGF-β and WNT signaling pathways in the pathogenesis of cardiac fibrosis. Although the cell biology of the TGF-β and WNT pathways has been well-described in cellular development and in the pathophysiology of many diseases, the mechanisms of these pro-fibrotic pathways in cardiac pathologies is less well-understood. As discussed in this review, canonical WNT signaling and TGF-β signaling can combine together to regulate fibrotic processes in the heart, and is likely to play a key role in switching on the genetic machinery for the pro-fibrotic changes. ncRNAs have increasingly been recognized to play possible roles in strategies for combating CVDs, as described above. Recent studies in the ncRNA field that are described in this review, also indicate the important function of ncRNAs in the regulation of cell signaling pathways, especially TGFB and WNT signaling. Information gathered from these experiments, and identification of the signaling pathways of the ncRNAs involved with cardiac fibrosis, may lead to a new target for treatment strategies for cardiac fibrosis. Additional research is needed to identify the exact details of the mechanism by which the ncRNA network affects cardiac fibrosis through TGFβ/WNT signaling. Moreover, the possible clinical importance of these TGFβ/WNT related ncRNAs, such as the use of microRNAs as therapeutic tools, and circRNAs as diagnostic/prognostic biomarkers for cardiac fibrosis should be examined in additional animal and clinical trials.

## Data Availability

The primary data for this study is available from the authors on direct request.

## Abbreviations

ACE2: Angiotensin-Converting Enzyme 2
ACTA2: Smooth muscle alpha (α)-2 actin
ADAM17: A disintegrin and metalloproteinase 17
ALDH2: Aldehyde dehydrogenase-2
AngII: Angiotensin II
AP-1: Activator protein 1
APC: Adenomatous polyposis coli
ATFs: Activating transcription factors
cAMP: response element modulator
CBP: CREB binding protein
CFs: Cardiac fibroblasts
CHF: Chronic heart failure
ChIP: Chromatin immunoprecipitation
CircHIPK3: Circular RNA Homeodomain Interacting Protein Kinase 3
circRNAs: Circular long noncoding RNAs
CK1: Casein kinase 1
CMR: Cardiac magnetic resonance
Crnde: Colorectal neoplasia differentially expressed
CTGF: Connective tissue growth factor
DCM: Dilated cardiomyopathy
DKK: Dikkopf
sFRP: Secreted frizzled-related protein
DVL: Dishevelled;
ECM: Extracellular matrix
ED-A: Extra domain A
Erk1/2: Extracellular signal-regulated kinase 1 and 2
FGF 23: Fibroblast growth factor 23
Fz: Frizzled
LRP: Low-density-lipoprotein-receptor-related proteins
GRB-2: Growth factor receptor-bound protein 2
GSK-3β: Glycogen synthase kinase-3β
HF: Heart failure
HOTAIR: HOX transcript antisense RNA
HOXA11-AS: Homeobox A11 antisense
HuR: Human antigen R
I/R: Ischemic-reperfusion (I/R) injury
I-Smad: Inhibitory Smad
ISO: Isoproterenol
SFRP5: Secreted frizzled-related protein 5
JNK: Jun N-terminal kinase
JNKs: c-Jun N-terminal kinases
LAP: Latency-associated protein
lncRNAs: Long noncoding RNAs
LTBPs: Latent TGF-β–binding proteins
LV: Left ventricle
MAPK: Mitogen-Activated Protein Kinase
MI: Myocardial infarction
MIAT: Myocardial infarction associated transcript
MiRNAs: MicroRNAs
mTOR: Mammalian target of rapamycin
ncRNAs: non-coding RNAs
NF-Κb: Nuclear factor kappa-light-chain-enhancer of activated B cells
NOD1: Nucleotide-binding oligomerization domain-containing protein 1
PAI: Plasminogen activator inhibitor
PI3K/Akt: Phosphatidylinositol 3-kinase/ Protein Kinase B
PKC: Protein kinase C
QSG: Qishen Granule
RAS: Renin-angiotensin system
ROCK: Rho-associated protein kinase
RSBEs: RNA Smad-binding elements
RTK: Receptor tyrosine kinase
SIRT3: Sirtuin 3 α-SMA Alpha-smooth muscle actin
STAT3: Signal Transducer and Activator of Transcription 3
TAC: Transverse aorta constriction
TAC:EphrinB2: Erythropoietin-producing hepatoma interactor B2
TCF/Lef-1: T-cell factor/lymphoid enhancer-binding factor-1
TG: Transgenic
LAD: Left anterior descending
TGF-β R1: Transforming growth factor beta receptor 1
TGF-β: Transforming growth factor beta
TIMP3: Tissue inhibitor of matrix metalloproteinases-3
TIMPs: Tissue inhibitor of metalloproteinases
TP53INP1: Tumor protein p53-inducible nuclear protein1
TRPA1: Transient receptor potential ankyrin 1
TUG1: Taurine Upregulated Gene 1
URI1: Unconventional prefoldin RPB5 interactor 1
WNT: Wingless-related integration site
XG: Xinfuli Granule
